# Accuracy of zygomatic dental implant placement using computer-aided static and dynamic navigation systems compared with a mixed reality appliance. An in vitro study

**DOI:** 10.4317/jced.61097

**Published:** 2023-12-01

**Authors:** Juan-Ramón González-Rueda, Agustín Galparsoro-Catalán, Víctor-Manuel de Paz-Hermoso, Elena Riad-Deglow, Álvaro Zubizarreta-Macho, Jesús Pato-Mourelo, Sofía Hernández-Montero, Javier Montero-Martín

**Affiliations:** 1 Department of Implant Surgery, Faculty of Health Sciences, Alfonso X el Sabio University, 28691 Madrid, Spain; 2 Department of Maxillofacial Surgery. Quiron Health Hospital. 28002 Madrid, Spain; 3 Department of Surgery, Faculty of Medicine, University of Salamanca, 37008 Salamanca, Spain; 4 Department of Surgery, Faculty of Dentistry, University of Navarra, 31009 Pamplona (Navarra), Spain

## Abstract

**Background:**

Analyze and compare the accuracy of zygomatic dental implant placement carried out using a static navigation surgery, a dynamic navigation surgery and an augmented reality appliance.

**Material and Methods:**

Eighty (80) zygomatic dental implants were randomly assigned to one of four study groups: A: static navigation implant surgery (n = 20) (GI); B: dynamic navigation implant surgery (n = 20) (NI); C: augmented reality appliance implant placement (n = 20) (ARI) and D: free hand technique (n = 20) (FHI). A preoperative cone-beam computed tomography (CBCT) scan of the existing situation was performed to plan the surgical approach for the computer assisted implant surgery study groups. Four zygomatic dental implants were placed in anatomical-based polyurethane models (n = 20) manufactured by stereolithography, and a postoperative CBCT scan was taken. Subsequently, the preoperative planning and postoperative CBCT scans were uploaded to dental implant software to analyze the coronal global, apical global, and angular deviations. Results were analyzed using linear regression models with repeated measures to assess the differences according to the group, according to the position, and the interaction between both variables. If statistically significant differences were detected, 2-to-2 comparisons were made between the groups/positions.

**Results:**

The results did not show statistically significant differences between the coronal global deviations of GI (5.54 ± 1.72 mm), NI (5.43 ± 2.13 mm), ARI (5.64 ± 1.11 mm) and FHI (4.75 ± 1.58 mm). However, showed statistically significant differences between the apical global deviations of FHI (3.20 ± 1.45 mm) and NI (4.92 ± 1.89 mm) (*p* = 0.0078), FHI and GI (5.33 ± 2.14 mm) (*p* = 0.0005) and FHI and ARI (4.88 ± 1.54 mm) (*p* = 0.0132). In addition, the results showed also statistically significant differences between the angular deviations of FHI (8.47º ± 4.40º) and NI (7.36º ± 4.12º) (*p* = 0.0086) and between GI (5.30º ± 2.80º) and ARI (9.60º ± 4.25º) (*p* = 0.0005).

**Conclusions:**

Free-hand technique provides greater accuracy of zygomatic dental implant placement than computer-assisted implant surgical techniques, and zygomatic dental implants placed in the anterior region are more accurate than in the posterior region. However, it is an *in vitro* study and further clinical studies must be conducted to extrapolate the results to the clinical setting.

** Key words:**Implantology, computer assisted implant surgery, image-guided surgery, augmented reality, navigation surgery, zygomatic implants.

## Introduction

Rehabilitation of severely atrophied, completely edentulous maxillae is of the utmost concern, and these procedures constitute a challenge for dental practitioners given the lack of available bone, which inhibits placement of dental implants of conventional length (1). Multiple alternative therapeutic procedures have been proposed as alternatives for the rehabilitation of atrophic maxilla, using bone augmentation techniques to increase bone availability and enable subsequent implant-supported rehabilitation, including sinus lifts, grafting procedures, and apposition grafts (with or without LeFort I osteotomies), with reported success rates ranging from 60–90% (2-4). However, most of these techniques necessitate delayed approaches and procedures of two stages or more, among them bone grafts, which heighten the potential risk of postoperative complications (5). Furthermore, the limited bone availability and/or low-inadequate bone density of edentulous patients with atrophic maxilla is linked to higher implant failure rates (6,7). As a result, some clinicians have proposed zygomatic dental implants as an alternative for rehabilitating fully edentulous maxillae without the need for bone grafting procedures (8). This rehabilitation technique using zygomatic implant has been employed in patients presenting with severe maxilla resorption, together with the use conventional-length dental implants, with reported survival rates ranging from 96–100% (9-11). Regrettably, there is also a risk of potential postoperative complications that may affect the maxillary sinus, particularly in the case of intrasinus zygomatic dental implant placement. Incidence of sinusitis has been reported in as many as5–6% of cases (range: 0-26.6%); that being said, therapy with antibiotics has also been demonstrated as widely effective in all patients (12,13). Moreover, research has also found prosthetic complications in implant-supported restorations that use zygomatic dental implants; these include effects on the elements of retention of overdentures, overgrowth of the mucosa, fractured fixed dental prostheses, hyperplasia, and discomfort (14). Additionally, clinicians have reported intraoperative and postoperative complications including fracture of the zygoma, infection, burning sensations, implant fenestration, and discomfort due to an implant protruding under the lower eyelid (15,16). It is therefore important to improve the accuracy of zygomatic dental implants to reduce the potential complications of intraoperative and postoperative dental implants, especially in extremely atrophic edentulous maxillae. In addition, this computer assisted implant surgery techniques based on cone-beam computed tomography (CBCT) scans have shown to also improve the accuracy of transferring the prosthetic digital planning to the jaw (17). In recent years, the field of dental surgery has seen the use of image data based navigation techniques for the placement of dental implants to improve procedure outcomes and avoid the potential risks carried by this therapeutic procedure (17). This alternative surgical approach is developed using preoperative CBCT scans and specialized software for 3D implantplanning, enabling more accurate placement of implants (18). In general, there are two different kinds of surgical implant placement techniques using computer assisted implant surgery (CAIS) techniques: dynamic (d-CAIS) and static (s-CAIS). Vrielinck *et al*. used a surgical template based on a preoperative CT scan to improve the accuracy of zygomatic dental implant placement and thereby increase survival rates (19). Chow *et al*. used this surgical protocol to enable immediate occlusal loading of zygomatic dental implants (20). d-CAIS systems can detect and tracking the position of optical reference markers, which are laid over the patient and surgical instruments via a tracking system array. Both CAIS techniques have been extensively studied, with findings showing that they enable highly accurate placement of dental implants (21-23). The mean horizontal deviation of s-CAIS systems at the coronal global and apical global are 1.2 mm (1.04–1.44 mm) and 1.4 mm (1.28–1.58 mm), respectively, with a mean angular deviation of 3.5° (3.0-3.96°) (24). Research has found that d-CAIS systems result in 0.81 mm deviation at the coronal global 0.91 mm deviation at the apical global and an angular deviation of 3.807º (25). The values of these findings have yet to be compared.

Augmented reality devices have also been used to improve visualization (26) and experimentally improve the accuracy of implant placement with conventional length dental implants (27); however, there is little published literature regarding these techniques, and clinical trials are needed to better evaluate the accuracy of these technologies. Augmented reality appliances have not been used before in the field of dental implants, and they could prove useful due to their accurate tracking technology and small size.

The objective of the present *in vitro* study was to evaluate and compare the accuracy of placement of zygomatic dental implants using a static navigation surgery, a dynamic navigation surgery, an augmented reality appliance and a free-hand approach on surgical models. The null hypothesis (H0) states that there is no difference in accuracy when comparing placement of zygomatic dental implants using a static navigation surgery, a dynamic navigation surgery, and an augmented reality appliance.

## Material and Methods

-Study Design

Researchers conducted a randomized controlled experimental trial in keeping with the principles outlined by the International Organization for Standardization (ISO 14801). This experimental trial was carried out between January to March 2021 at the Dental Centre of Innovation and Advanced Specialties at the Alfonso X El Sabio University (Madrid, Spain). The Ethical Committee of the Alfonso X El Sabio University approved the study in December 2020 (process no. 23/2020). The patient provided their informed consent to for their preoperative cone-beam computed tomography (CBCT) scan to be used in this study.

Eighty (80) zygomatic dental implants (Galimplant, Sarria, Lugo, Spain) were planned and placed in teeth in positions 2.4 (4.3 mm × 30 mm, internal taper and conical wall), 2.2 (4.3 mm × 50 mm, internal taper and conical wall), 1.2 (4.3 mm × 52,5 mm, internal taper and conical wall), and 1.4 (4.3 mm × 35 mm, internal taper and conical wall). Researchers used an ANOVA to establish the sample size, achieving 80% power with a confidence level of 5%, with a variability between groups of 0,6 and variability intra groups of 4, to identify differences in the contrast of the null hypothesis H0: μ1 = μ2 = μ3 = μ4.

Researchers randomized the zygomatic dental implants (Epidat 4.1, Galicia, Spain), which were assigned to one of the following study groups: A:zygomatic dental implants (Galimplant, Sarria, Lugo, Spain), with placement using a s-CAIS system (NemoStudio®, Nemotec, Madrid, Spain) (n = 20) (guided implant (GI)); B, zygomatic dental implants (Galimplant, Sarria, Lugo, Spain), with placement using a d-CAIS (Navident, ClaroNav, Toronto, Canada) (n=20) (navigation implant (NI)); C, zygomatic dental implants (Galimplant, Sarria, Lugo, Spain), with placement using a augmented reality appliance(Hololens1, Redmond, WA, USA) (n = 20) (augmented reality implants (ARI)); and D, zygomatic dental implants (Galimplant, Sarria, Lugo, Spain), which were placed manually using a freehand technique (n = 20) (freehand implant (FHI)). The order of placement of the zygomatic dental implants (Galimplant, Sarria, Lugo, Spain) was randomized across the study groups (Epidat 4.1, Galicia, Spain), beginning with the GI study group and followed by the NI study group, ARI study group, and FHI control group. Drilling order was also randomized (Epidat 4.1, Galicia, Spain), beginning with the zygomatic dental implant located in position 1.2 and followed by the zygomatic dental implant located in position 2.2, 1.4 and 2.4.

-Imaging and Fabrication of Surgical Models

Twenty (20) anatomically based standardized polyurethane models of a completely edenulous, atrophic upper jaw maxilla were manufactured using a3D impression procedure (Sawbones Europe AB, Malmo, Sweden) based on a preoperative CBC scan (WhiteFox, Satelec, Merignac, France). The scan was taken from a real patient using the following exposure parameters: 8.0mA, 105.0 kV peak, 7.20 s, with a field of view of 15 mm × 13 mm.

-Surgical Planning

**Figure 1:**
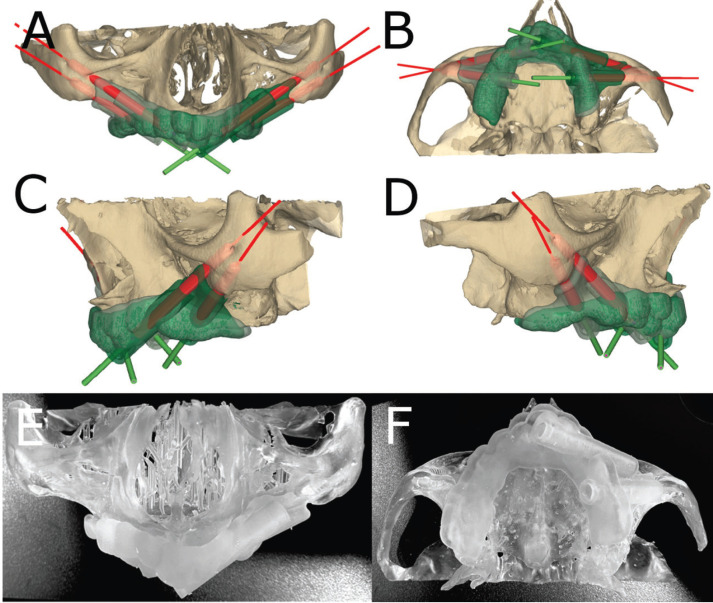
(A) Front, (B) bottom, (C) left, and (D) right lateral views of the dental implants and virtual template planning. (E) Front and (F) bottom view of the surgical template adjustment on the experimental models.

The zygomatic dental implants (Galimplant, Sarria, Lugo, Spain) from the GI study group were virtually planned using 3D implant-planning software (NemoScan, Nemotec, Madrid, Spain) with the aforementioned measurements (Fig. 1A,B). After the virtual templates were designed (Fig. 1A,D), they were manufactured using stereolithography (ProJet 6000, 3D Systems, Rock Hill, SC, USA) for a half guided surgical procedure at 1000rpm under cooling, following the manufacturer recommendations (Fig. 1E,F). These templates were apt for the experimental models, with no further adjustments being made.

**Figure 2:**
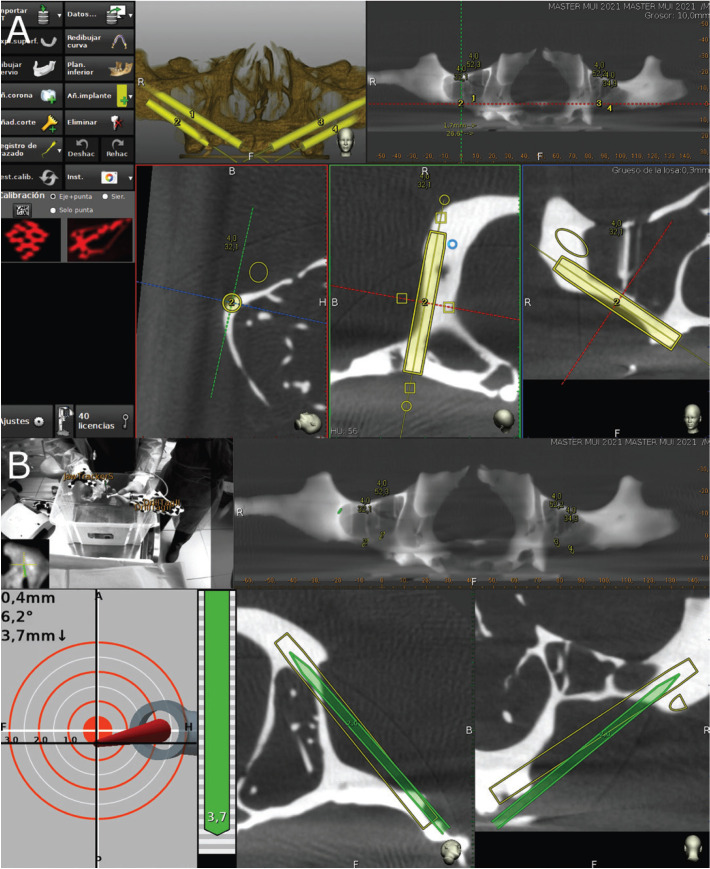
(A) Treatment-planning software preoperative planning of placement of the zygomatic dental implant for the dynamic navigation surgery appliance and (B) tracking procedure during zygomatic dental implant placement.

A preoperative CBCT scan was taken of the NI anatomical-based standardized polyurethane models (WhiteFox, Satelec, Merignac, France) prior to placing a jaw tag, which allow the recognition and planning procedures. This black and¬ white tag was affixed to the dental surface of the anatomically based, standardized polyurethane models using a photo-polymerized composite resin. Afterwards, the STL digital file from the surgical planning performed with the 3D implant-planning software (NemoScan, Nemotec, Madrid, Spain), was imported to the treatment-planning software (Navident, ClaroNav, Toronto, Canada) of the d-CAIS to guide the surgical planning. The datasets obtained from the CBCT scan were imported into a treatment-planning software (Navident, ClaroNav, Toronto, Canada) on a laptop computer mounted on a mobile unit to simulate placement of the zygomatic dental implant like the prior surgical planning (Fig. 2A). Another B&W drill tag was attached to the handpiece (W&H, Bürmoos, Austria). Researchers calibrated and identified both optical reference markers using an optical triangulation tracking system with stereoscopic motion-tracking cameras, which oriented the drilling process in real time, ensuring the planned angle, pathway, and depth were achieved. A zygomatic dental implant system (Galimplant, Sarria, Lugo, Spain) was used to perform the drilling, with this procedure being monitored on the laptop computer with the d-CAIS system (Fig. 2B).

-Composition of the AR system

The zygomatic dental implants (Galimplant, Sarria, Lugo, Spain) that had been randomly assigned to the ARI study group were virtually planned using a 3D implant-planning software (NemoScan, Nemotec, Madrid, Spain) with the aforementioned measurements. The STL digital file of the dental implants positioning was uploaded to an augmented reality appliance (Hololens1, Microsoft, Redmond, WA, USA), to enable drilling in all space planes (INNOAREA, Valencia, Spain) without any delay related to the detection and tracking of the anatomical-based standardized polyurethane models. The information related to the position of the zygomatic dental implants was virtually transferred to the anatomical-based standardized polyurethane models (WhiteFox, Satelec, Merignac, France) by showing the zygomatic dental implants as three-dimensional cylinders. Tracking was achieved by four tracking cameras incorporated in the augmented reality appliance (Hololens1, Microsoft, Redmond, WA, USA) (27).

The zygomatic dental implants (Galimplant, Sarria, Lugo, Spain) that had been randomly assigned to the FHI control group were placed manually, with access to CBCT scan and the preoperative planning (NemoStudio®, Nemotec, Madrid, Spain). All zygomatic dental implants (Galimplant, Sarria, Lugo, Spain) were placed by a unique operator with prior surgical experience who had received training sessions in static CAIS, dynamic CAIS and augmented reality surgical techniques for zygomatic dental implant placement.

-Data Acquisition

**Figure 3:**
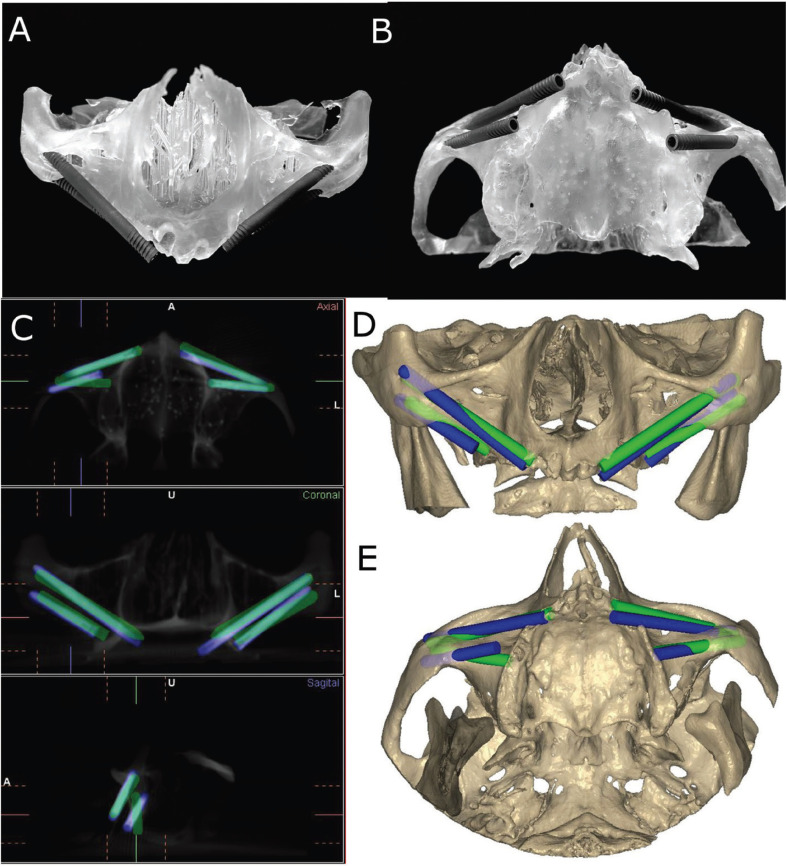
(A) Front and (B) bottom view of the zygomatic dental implants placed on the experimental model. (C) CBCT images, (D) front and (E) bottom view of the measurement procedure between the preoperative planning (green cylinders) and postoperative zygomatic dental implant placement (blue cylinders).

Following placement of the zygomatic dental implant, researchers conducted postoperative CBCT scans (WhiteFox, Satelec, Merignac, France) using the exposure parameters. The planning and postoperative CBCT scans (WhiteFox, Satelec, Merignac, France) of the different study groups were subsequently uploaded into a 3D implant-planning software (NemoScan, Nemotec, Madrid, Spain). The scans were then matched to assess the coronal global deviation, measured at the coronal entry point (mm), apical global deviation (mm), and angular deviation (°), with the latter measured in the center of the cylinder. Any deviations that were noted in any of the implants were subsequently evaluated and compared between axial, sagittal, and coronal views (Fig. 3A-E) by an independent operator. In addition, the deviations in the position of zygomatic dental implant positions were also recorded and analyzed.

-Statistical Analysis

For each of the response variables, Tables have been obtained with summary statistics based on group, position, and group and position: number of observations, mean, standard deviation, median, and minimum and maximum values. They have been represented graphically using box plots.

Linear regression models with repeated measures have been adjusted to analyze the differences according to the group, according to the position, and the interaction between both variables. If statistically significant differences were detected, 2-to-2 comparisons were made between the groups/positions. The *p-value*s have been adjusted using the Tukey method to correct type I error.

Statistical analysis was performed with the software: SAS v9.4, SAS Institute Inc., Cary, NC, USA. The statistical decisions have been made taking the value 0.05 as the level of significance.

## Results

Table 1 shows the means, medians and SD values for the coronal global (mm), apical global (mm) and angular deviations (°) of the GI, NI, ARI, and FHI study groups.

**Table 1:**
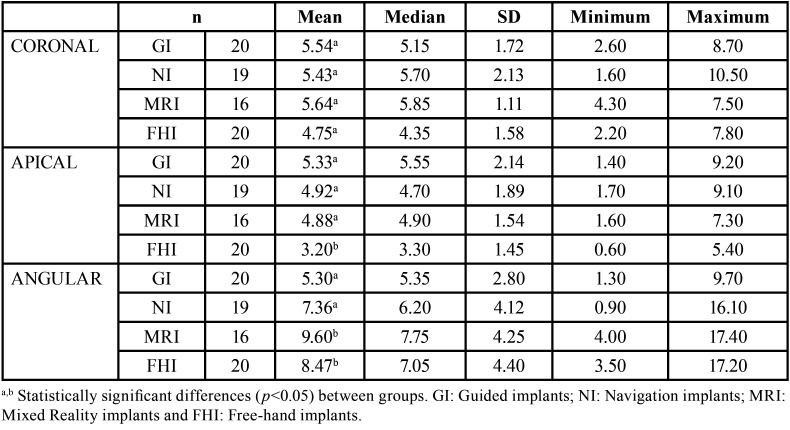
Descriptive values for the deviation at the coronal entry point (mm), apical endpoint (mm) and angular (°) deviations of (guided implant (GI) using a static navigation system; navigation implant (NI) using a dynamic navigation system).

**Figure 4:**
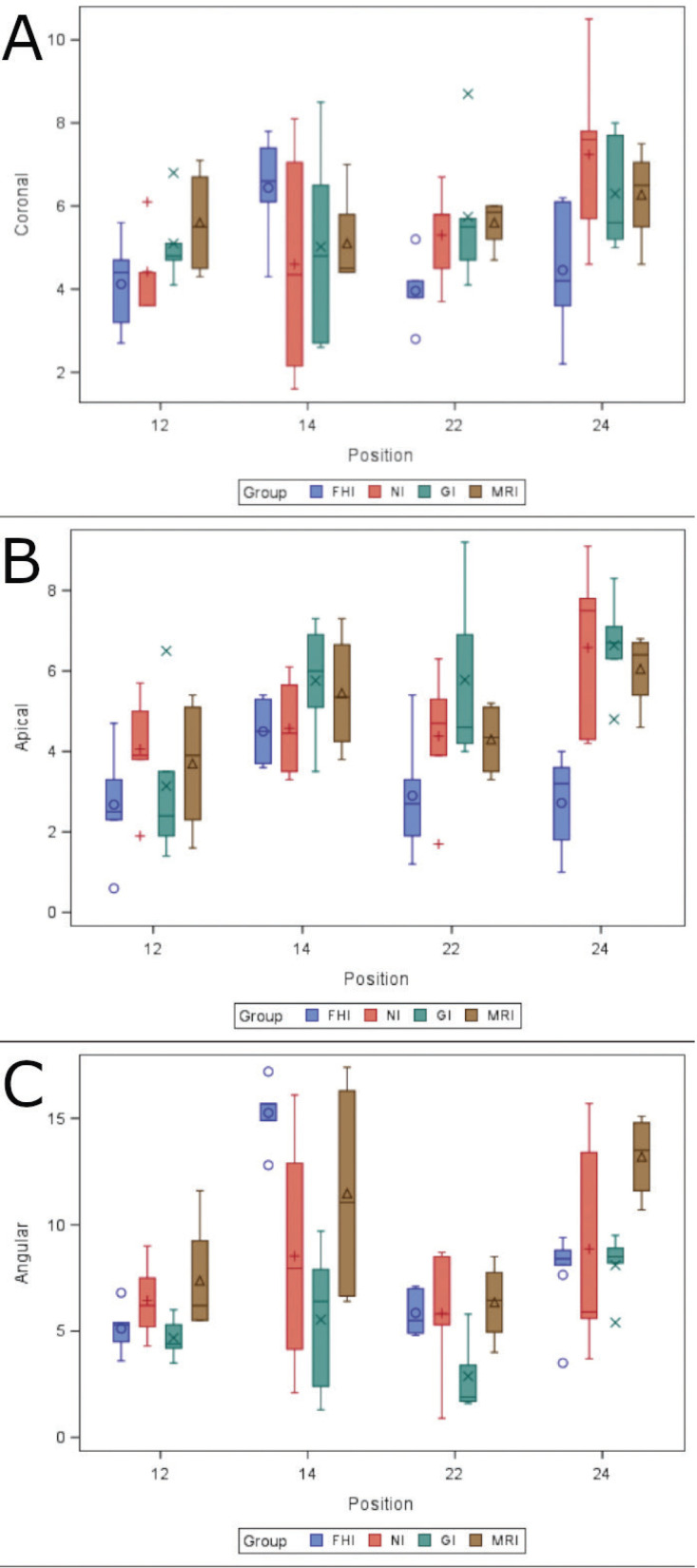
(A) Box plot of deviations at the coronal, (B) apical and (C) angular deviations in the study groups and zygomatic dental implant positions. Median values are represented by the horizontal lines in each box.

We did not find any statistically significant differences between the study groups in coronal global deviations (*p* = 0.2904), nor in the zygomatic dental implant positions (*p* = 0.1068) (Fig. 4A). However, there were statistically significant differences found between zygomatic dental implant positions 1.2 (4.77 ± 1.18 mm) and 2.4 (6.06 ± 1.90 mm) of the NI study group (*p* = 0.033).

We found statistically significant differences in the apical global deviations of the FHI (3.20 ± 1.45 mm) control group and NI (4.92 ± 1.89 mm) study group (*p* =0.0078), FHI control group and GI (5.33 ± 2.14 mm) study group (*p* = 0.0005), and FHI control group and ARI (4.88 ± 1.54 mm) study group (*p* = 0.0132). However, no statistically significant differences were found between the NI and GI study groups (*p* = 0.8309), NI and ARI study groups (*p* = 1.0000), and GI and ARI study groups (*p* = 0.8278). In addition, statistically significant differences were found between zygomatic dental implant positions 1.2 (3.38 ± 1.64 mm) and 1. 4 (5.08 ± 1.33 mm) (*p* = 0.0117) and 1.2 and 2. 4 (5.47 ± 2.21 mm) (*p* = 0.0008). In addition, the GI study group showed higher deviations at the apical global between zygomatic dental implant positions 1.2 and 2. 4 (*p* = 0.0050), resulting in a statistically significant difference between zygomatic dental implants placed in position 2.4 of the FHI control group and NI study group (*p* = 0.0017), FHI control group and GI study group (*p* = 0.0014), and FHI control group and ARI study group (*p* = 0.0140) (Fig. 4B).

We also found statistically significant differences between angular deviations of the study groups (*p* = 0.0006) and zygomatic dental implant positions (*p* <0.001), even detecting a relationship between the study group and zygomatic dental implant position (*p* = 0.0165). Statistically significant differences were observed between the FHI (8.47 ± 4.40º) control group and NI (7.36 ± 4.12º) study group (*p* = 0.0086) and between the GI (5.30 ± 2.80º) and ARI (9.60 ± 4.25º) study groups (*p* = 0.0005). In addition, statistically significant differences were found between zygomatic dental implant positions 1.2 (5.83 ± 1.96º) and 1.4 (10.22 ± 5.52º) (*p* = 0.0004), 1.2 and 2.4 (9.25 ± 3.66º) (*p* = 0.0038), 1.4 and 2.2 (5.17 ± 2.41º) (*p* <0.0001), and 2.2 and 2.4 (*p* = 0.0004), particularly between the FHI control group and ARI study group (Fig. 4C).

One zygomatic dental implant and four dental implants were withdrawn from the NI and ARI study groups, respectively, because the osteotomy site preparations did not provide sufficient stability for the zygomatic dental implants.

## Discussion

The results of the present study reject the null hypothesis (H0), which states that there is no difference in the accuracy of zygomatic dental implants placed using a s-CAIS system, a d-CAIS system, and an augmented reality appliance.

The results of the present study show that the conventional free-hand technique provides greater accuracy in the placement of zygomatic dental implants at the coronal and apical level than the static computer assisted implant surgery technique, dynamic computer assisted implant surgery technique, or augmented reality techniques; however, the static computer assisted implant surgery technique resulted in less angular deviation than the dynamic computer assisted implant surgery technique, augmented reality technique, or the free-hand control group. In summary, the FHI approach showed lower deviation values at the coronal global and apical global. This may be because the zygomatic dental implants assigned to the FHI control group were the last to be placed, which meant the operator was able to learn and memorize the correct position of the zygomatic dental implants. Furthermore, zygomatic dental implants placed in posterior regions showed higher deviation values at the coronal global, apical global, and angular level. Moreover, the zygomatic dental implants located in the anterior region showed less horizontal and angular deviation than posterior dental implants, perhaps due to better accessibility and visibility.

Static computer assisted implant surgery techniques have been studied for use in placement of zygomatic dental implants. Vrielinck *et al*. reported a mean coronal global deviation of 2.77 ± 1.61 mm, a mean apical global deviation of 4.46 ± 3.16 mm, and a mean angular deviation of 5.14 ± 2.59° (19). However, the accuracy of these s-CAIS techniques has been widely studied when using conventional length dental implants, finding a mean coronal global horizontal deviation and apical global deviation of 1.2 mm (1.04-1.44 mm) and 1.4 mm (1.28-1.58 mm), respectively, with a mean angular deviation of 3.5° (3.0-3.96°) (25). In addition, dynamic computer assisted implant surgery techniques have not been used for the placement of zygomatic dental implants, nor have they been compared with the accuracy of s-CAIS techniques, despite having shown to be highly accurate when used with conventional length dental implants. Stefanelli *et al*. found a mean coronal global deviation of 0.71 ± 0.40 mm, a mean apical global deviation of 1.00 ± 0.49 mm, and a mean angular deviation of 2.26 ± 1.62° (28). Hoffmann *et al*. found statistically significant differences between d-CAIS systems and manual implant placement regarding the accuracy of implant placement, with mean angular deviations of 4.2 ± 1.8° and 11.2 ± 5°, respectively (29). When using a d-CAIS system (1.35 ± 0.55 mm), a s-CAIS system (1.50 ± 0.79 mm), and manual implant placement (2 ± 0.79 mm), Chen *et al*. reported similar mean horizontal deviation values at the apical global deviation. Higher values of angular deviation values were found when using a d-CAIS system (4.45 ± 1.97°), s-CAIS system (6.02 ± 3.71°), and manual implant placement (9.26 ± 3.62°) (30). However, s-CAIS and d-CAIS techniques did not result in statistically significant differences at the coronal (*p* = 0.2904), apical (*p* = 0.8309), and angular level (*p* = 0.1410) in the present study, perhaps because the d-CAIS systems have a steep learning curve that could influence results (25). The results of the present study are aligned with the study by Mediavilla-Guzman *et al*., who compared the accuracy of s-CAIS and d-CAIS systems in the placement of conventional length dental implants and reported no statistically significant differences between s-CAIS and d-CAIS systems at the coronal (*p* = 0.6535) and apical (*p* = 0.9081) levels. They did observe statistically significant differences between the angular deviations of s-CAIS and d-CAIS systems (*p*= 0.0272) (23). The results of the present study found higher horizontal deviations (especially at the apical global deviation) due to the longer dental implant length of zygomatic dental implants. The accuracy of dental implant placement using s-CAIS techniques directly depends on the design and manufacturing process of the surgical template; in other words, inaccurate manufacturing could lead to intraoperative complications (17). However, d-CAIS systems prevent the clinician to have a direct view of the surgical field, although give them the ability to reorient an implant’s position, if necessary.25,26 Furthermore, these systems prove particularly useful for patients with limited mouth openings or in treatments of the posterior region (23-27). The primary drawback of d-CAIS systems is how difficult it is to keep sight of the d-CAIS system display throughout the procedure. Therefore, augmented reality devices are often used to display a virtual image of the d-CAIS system while maintaining visibility of the surgical field (31,32). The accuracy rates of image-guided navigation systems are comparable regarding the depth, position, and angle of implants. These factors are necessary to prevent intraoperative surgical complications and ensure proper positioning of dental implants, as poor positioning can compromise primary stability and inhibit the use of immediate-loading restoration techniques (26,27,29). In addition, these techniques also circumvent the need for wide excisions often required to expose the implant platform after healing is completed, enabling a minimally invasive, transgingival approach for implant placement (24,29). These techniques may be especially helpful for use with high-risk patients, including cardiovascular patients who take anticoagulation medications, or those with atrophic, edentulous jaws (29).

Augmented reality technology has been used in the education industry and by manufacturers for improving dental education, particularly in the field of dental implants (26); in addition, augmented reality devices have also been used to visualize maxillectomy defects. However, a target image with a symbol track marker is needed to superimpose the virtual models over the real case with augmented reality application (27). This limitation was solved in the present study by improving the tracking method without track markers. Furthermore, Ma *et al*. assessed experimentally the accuracy of augmented reality technology for dental implant placement, reporting a mean target error between 1.25 and 1.63 mm, and a mean angle error between 4.03° and 6.10° (33). Jiang *et al*. found a mean horizontal deviation of< 1.5 mm and a mean angular deviation of < 1.5° (34). Additionally, Kivovics *et al*. reported no statistically significant differences between the dental implant position of conventional length dental implants at coronal global deviation (1.27 ± 0.40 mm and 1.31 ± 0.42 mm), apical global deviation (1.34 ± 0.41 mm and 1.38 ± 0.41 mm) and angular deviation (4.09 ± 2.79° and 3.21 ± 1.52°) between augmented reality technology and s-CAIS (35). However, these studies were performed with conventional length dental implants without comparing the positioning of dental implants with a d-CAIS. In addition, the angular deviation of this previous studies showed lower angular deviation, due to the higher length and tilted nature of the zygomatic dental implants are a challenge to the operator for the positioning at coronal, apical and angular level, comparing to convention length dental implants. However, an augmented reality technology combining augmented and virtual reality has yet to be studied for the placement of dental implants. The present study found that the placement of zygomatic dental implants using augmented reality device resulted in higher coronal and angular deviations when compared with s-CAIS and d-CAIS study groups, as well as the free-hand control group; however, operators felt that the techniques were very promising, and further studies are recommended.

The strength of this study is that there is no previous study in literature that compares the accuracy of static computer assisted implant surgery, dynamic computer assisted implant surgery, augmented reality technology and conventional free-hand approach for zygomatic dental implant placement and the need for the study is obviously that the accuracy of new surgical modalities must be tested against more established navigational methods. This *in vitro* study is somewhat limited in scope due to its experimental nature. The methodological procedure used in this study is easily applicable to clinical studies; however, the improvements performed in augmented reality appliances will provide most accurate results in the future. Moreover, the order of navigation methods may have caused learning by the time the operator reached the free hand placement. Additionally, the measurement analysis of the zygomatic dental placement between the navigation techniques was performed in basis of CBCT scans instead of STL digital files, which has proven to be more accuracy (3). Furthermore, the surgical template was not stablished by pins or screws, which could have influenced the accuracy of the zygomatic dental implants placed with s-CAIS.

## Conclusions

Bearing in mind the limitations of the present study, its results found that the manual free-hand technique provides greater accuracy in the placement of zygomatic dental implants than computer assisted implant surgical techniques. In addition, placement of zygomatic dental implants in the anterior region is more accurate than in the posterior region.
